# VEGFR1-Positive Macrophages Facilitate Liver Repair and Sinusoidal Reconstruction after Hepatic Ischemia/Reperfusion Injury

**DOI:** 10.1371/journal.pone.0105533

**Published:** 2014-08-27

**Authors:** Hirotoki Ohkubo, Yoshiya Ito, Tsutomu Minamino, Koji Eshima, Ken Kojo, Shin-ichiro Okizaki, Mitsuhiro Hirata, Masabumi Shibuya, Masahiko Watanabe, Masataka Majima

**Affiliations:** 1 Department of Pharmacology, Kitasato University School of Medicine, Sagamihara, Kanagawa, Japan; 2 Department of Surgery, Kitasato University School of Medicine, Sagamihara, Kanagawa, Japan; 3 Department of Gastroenterology, Kitasato University School of Medicine, Sagamihara, Kanagawa, Japan; 4 Department of Immunology, Kitasato University School of Medicine, Sagamihara, Kanagawa, Japan; 5 Gakubunkan Institute of Physiology and Medicine, Jobu University, Takasaki, Gunma, Japan; Leiden University Medical Center, Netherlands

## Abstract

Liver repair after acute liver injury is characterized by hepatocyte proliferation, removal of necrotic tissue, and restoration of hepatocellular and hepatic microvascular architecture. Macrophage recruitment is essential for liver tissue repair and recovery from injury; however, the underlying mechanisms are unclear. Signaling through vascular endothelial growth factor receptor 1 (VEGFR1) is suggested to play a role in macrophage migration and angiogenesis. The aim of the present study was to examine the role of VEGFR1 in liver repair and sinusoidal reconstruction after hepatic ischemia/reperfusion (I/R). VEGFR1 tyrosine kinase knockout mice (VEGFR1 TK^-/-^ mice) and wild-type (WT) mice were subjected to hepatic warm I/R, and the processes of liver repair and sinusoidal reconstruction were examined. Compared with WT mice, VEGFR1 TK^-/-^ mice exhibited delayed liver repair after hepatic I/R. VEGFR1-expressing macrophages recruited to the injured liver showed reduced expression of epidermal growth factor (EGF). VEGFR1 TK^-/-^ mice also showed evidence of sustained sinusoidal functional and structural damage, and reduced expression of pro-angiogenic factors. Treatment of VEGFR1 TK^-/-^ mice with EGF attenuated hepatoceullar and sinusoidal injury during hepatic I/R. VEGFR1 TK^-/-^ bone marrow (BM) chimeric mice showed impaired liver repair and sinusoidal reconstruction, and reduced recruitment of VEGFR1-expressing macrophages to the injured liver. VEGFR1-macrophages recruited to the liver during hepatic I/R contribute to liver repair and sinusoidal reconstruction. VEGFR1 activation is a potential therapeutic strategy for promoting liver repair and sinusoidal restoration after acute liver injury.

## Introduction

Ischemia/reperfusion (I/R) injury to the liver is a major complication of hemorrhagic shock, liver resection, and transplantation. Although hepatocytes are most susceptible, liver sinusoidal endothelial cells (LSEC) are also injured during hepatic I/R [1], [2], [3]. Hepatic I/R elicits tissue repair, a process of healing in the liver, which is characterized by the proliferation of hepatocytes, removal of necrotic tissue, and restoration of the hepatocellular and hepatic microvascular architecture. Hepatic tissue repair plays a critical role in determining the final outcome of hepatic I/R injury because a delay in liver repair and regeneration is associated with increased morbidity and mortality. However, the mechanisms underlying hepatocellular regeneration and sinusoidal restoration from hepatic I/R injury are unclear [4], [5].

Vascular endothelial growth factor (VEGF)-A is a major regulator of both vascular development and physiological and pathological angiogenesis during tumorigenesis, inflammation, and wound healing [6], [7]. The biological activity of VEGF-A is dependent on its interaction with specific receptors. VEGF acts primarily through two tyrosine kinase receptors: VEGF receptor-1 (VEGFR1) and VEGF receptor-2 (VEGFR2) [6], [7]. VEGF-induced angiogenesis is mediated primarily by VEGFR2, whereas VEGFR1 signaling contributes to pathological angiogenesis under certain conditions [8]–[10]. VEGFR1 is expressed on monocytes/macrophages [11] and plays an important role in macrophage recruitment to inflamed and cancerous tissues [12], [13].

VEGFR2 signaling is also required for liver regeneration following liver resection [14]. Genetic ablation of VEGFR2 impaired liver regeneration in a mouse model of partial hepatectomy, although the residual liver architecture remained intact [14]. VEGFR1 plays a role in liver repair in other models of liver regeneration, including hepatotoxic chemical-induced liver injury, which causes severe hepatocellular and microvascular damage. After carbon tetrachloride (CCl_4_) administration, VEGFR1 activation elicits the paracrine release of growth factors, resulting in hepatocyte proliferation in mice [15]. A similar role for VEGFR1 signaling in liver repair was demonstrated in a mouse model of acetaminophen hepatotoxicity [16]. During hepatic I/R injury, leukotriene B4 receptor 1 (BLT1) promotes liver repair via the recruitment of VEGFR1-expressing macrophages [17].

Although these studies indicate that VEGFR1 activation is crucial for liver repair after acute liver injury, it is not known whether VEGFR1 signaling is essential for liver repair and for restoration of the hepatic microvasculature after hepatic I/R injury. Therefore, the aims of the present study were to examine whether VEGFR1 signaling facilitates hepatocellular and sinusoidal repair after hepatic I/R and to identify the mechanism(s) underlying liver repair mediated by VEGFR1.

## Materials and Methods

### Animals

Male C57Bl/6 wild-type (WT) mice (8 weeks-of-age) were obtained from Crea Japan (Tokyo, Japan). VEGFR1 tyrosine kinase knockout mice (VEGFR1 TK^-/-^ mice, 8-weeks-old) with a C57BL/6 hybrid background were generated in-house [8]. For the BM transplantation experiments, transgenic mice expressing green fluorescent protein (GFP) against a C57BL/6 background were used to confirm BM chimerism. VEGFR1 TK^-/-^ mice and GFP+/+ mice were crossed to obtain GFP+VEGFR1 TK^-/-^ mice [18].

All animal experimental procedures were approved by the Animal Experimentation and Ethics Committee of the Kitasato University School of Medicine (2013–072), and were performed in accordance with the guidelines for animal experiments set down by Kitasato University School of Medicine.

### Model of liver ischemia-reperfusion

Animals underwent either sham surgery or I/R. Partial hepatic ischemia was elicited as previously described [17]. Briefly, mice were anesthetized with pentobarbital sodium (50 mg/kg, intraperitoneally (i.p.)). A laparotomy was performed and the blood supply to the median and left hepatic lobes was occluded for 1 h using an atraumatic vascular clamp. Reperfusion was initiated by removing the clamp. Sham control mice underwent the same protocol without vascular occlusion. In another set of experiments, some mice were injected i.p. with recombinant mouse epidermal growth factor (EGF) (10 µg/mouse) (AbD Serotec, Raleigh, NC) or PBS [19] at the time of clip removal and at 24 hours after reperfusion.

### Time course experiments

Mouse livers were subjected to ischemia for 60 min. Blood was drawn and livers were excised at 6, 24, 48, and 96 h after reperfusion. The serum was used to determine alanine aminotransferase (ALT) activity in a Dri-Chem 4000 Chemistry Analyzer System (Fujifilm, Tokyo, Japan). A part of the excised ischemic left lobe of the liver was fixed in phosphate-buffered formalin solution (10% v/v) and embedded in paraffin for histological evaluation.

### Bone marrow transplantation

Bone marrow (BM) transplantation was performed as previously described [18]. Briefly, donor BM cells from GFP+VEGFR1 TK^-/-^ mice and their GFP+WT counterparts were harvested using the same method [18]. Donor BM-derived mononuclear cells (2×10^6^ cells/200 µL PBS) were injected into the tail vein of irradiated WT mice. After 8 weeks, peripheral blood was collected and GFP expression was analyzed by fluorescence activated cell sorting (FACS) to assess BM chimerism. Mice in which more than 90% of the peripheral leukocytes were donor marker-positive were used for subsequent experiments.

### Histology and immunohistochemistry

Excised liver tissues were fixed immediately with 4% paraformaldehyde in 0.1 M sodium phosphate buffer (pH 7.4) for histological analysis [20]. Sections (4 µm thick) were prepared from paraffin-embedded tissue and subjected to either hematoxylin and eosin (H&E) staining or immunostaining. The level of necrosis (as a percentage of the total area) was estimated by measuring the necrotic area relative to the entire histological section, and an analysis of the necrotic area was performed with a VH analyzer (Keyence, Osaka, Japan). The hemorrhagic area was also determined to quantify the extent of hemorrhage. The results were expressed as a percentage. Sections were also stained for proliferating cell nuclear antigen (PCNA) (Invitrogen, Carlsbad, CA), and the levels measured. Each treatment group comprised five to six mice per time point. The number of PCNA-positive hepatocytes per 1000 hepatocytes was counted in six separate high power fields (×400) per animal. The percentage of PCNA-positive cells was then calculated and the results expressed as a PCNA-labeling index.

### Immunofluorescence staining

Tissue samples were fixed with periodate-lysine-paraformaldehyde (PLP) fixative at room temperature for 3 h. Following cryoprotection with 30% sucrose/0.1 M phosphate buffer (pH 7.2), sections (approximately 10 to 20 µm thick) were cut in a cryostat. Sections were then incubated with 1% bovine serum albumin (BSA)/PBS at room temperature for 1 h to block non-specific binding, followed by incubation with a rat anti-mouse F4/80 monoclonal IgG2a antibody, a macrophage marker including resident Kupffer cells (Santa Cruz Biotechnology Inc., Santa Cruz, CA), a rat anti-mouse CD11b monoclonal IgG2b antibody, a myeloid cell maker including recruited macrophages (AbD Serotec, Raleigh, NC), Ly6B, an anti-mouse neutrophil allotypic marker antibody, a neutrophil marker (AbD Serotec, Raleigh, NC) [21], a rabbit anti-mouse VEGFR1 polyclonal IgG antibody (Santa Cruz Biotechnology Inc., Santa Cruz, CA), a rat anti-mouse tyrosine kinase with immunoglobulin (Ig)G-like and endothelial growth factor-like domains 2 (Tie2) monoclonal antibody (LifeSpan Biosciences Inc., WA), a rabbit anti-mouse lymphatic vessel endothelial hyaluronan receptor (Lyve-1) antibody (Abcam, Cambridge, MA), a goat anti-mouse EGF antibody (R&D Systems, MN), and a rabbit phosphorylated histone H3 (pH 3) polyclonal antibody (Cell Signaling Technology, Inc., MA). After washing three times in PBS, the sections were incubated with a mixture of the following secondary antibodies for 1 h at room temperature: Alexa Fluor 488-conjugated donkey anti-rabbit IgG (Molecular Probes), Alexa Fluor 594-conjugated donkey anti-rat IgG (Molecular Probes), and Texas Red (TR)-conjugated donkey anti-goat IgG (Santa Cruz Biotechnology, USA). As a negative control, sections were incubated in 1% BSA-PBS in the absence of primary antibody. Images were captured under a fluorescence microscope (Biozero BZ-9000 Series; KEYENCE, JAPAN). After labeling, six low power optical fields (200× magnification) were randomly selected and the number of positive cells counted. At least five animals were analyzed per marker. Images were also captured with a confocal scanning laser microscope (LSM700; Zeiss, Jena, Germany), and computer assisted morphometric analyses were performed with ZEN 2009 software (Zeiss).

### Real-time RT-PCR

Transcripts encoding VEGF-A, VEGFR1, VEGFR2, interleukin (IL)-6, tumor necrosis factor-α (TNFα), hepatocyte growth factor (HGF), EGF, angiopoietin (Ang)-1, Ang-2, Tie2, and glyceraldehyde-3-phosphate dehydrogenase (GAPDH) were measured by real-time RT-PCR. Briefly, total RNA was extracted from liver tissues and homogenized in TRIzol reagent (Invitrogen, Carlsbad, CA). RNA expression was measured in a BioPhotometer (Eppendorf Co. Ltd., Tokyo, Japan). The primers used for real-time PCR were designed using Primer 3 software (http://primer3.sourceforge.net/) based on data from GenBank; the sequences are listed in Table S1. Data were normalized to the expression level of GAPDH in each sample.

### In vivo microscopy

Animals were anesthetized with pentobarbital sodium (50 mg/kg, intraperitoneally) and tissues were prepared for *in vivo* fluorescence microscopy as previously described [16], [22], [23]. The hepatic microcirculation was observed using a fluorescence microscope (ECLIPSE FN1, upright type; Nikon, Tokyo) fitted with a 100 W mercury lamp for epi-illumination. The microscopic images were obtained with an objective lens (20×/0.75 N.A.; Nikon) and images were recorded with a CCD camera (Evolve 512, Photometrics) and image analysis software (StreamPix, Norpix, Canada). Mice were injected intravenously with 50 µL of acetylated low density lipoprotein (Ac-LDL) (1∶2 dilution in PBS; Invitrogen) immediately prior to liver sinusoidal visualization [16]. Microvascular events were observed and recorded. The relative adequacy of blood perfusion through the sinusoids was evaluated by counting the number of sinusoids exhibiting blood flow in ten regions in each animal. The number of perfused sinusoids was expressed as a percentage of total sinusoids, regardless of blood flow per region.

### Cell culture

Peritoneal macrophages were induced in WT mice and VEGFR1 TK^-/-^ mice using thioglycollate [20]. Three days after i.p. injection of 2 ml of 4% thioglycollate medium (Nissui Pharmaceutical Co. Ltd, Tokyo, Japan), induced macrophages were obtained via peritoneal lavage with 2×5 ml PBS. Peritoneal exudate cells were washed and suspended in Roswell Park Memorial Institute (RPMI)-1640 medium containing 10% fetal bovine serum (FBS), 100 U/ml penicillin, and 100 µg/ml streptomycin in six-well tissue culture plates (2×10^6^ cells/well), and the macrophages were enriched by allowing adhesion for 1 h. The resulting peritoneal macrophages were plated in six-well tissue culture plates (3×10^5^ cells/well) and stimulated with VEGF-A (Acris Antibodies Inc., CA, USA).

Human umbilical vascular endothelial cells (HUVECs) (Kurabo, Tokyo, Japan) were cultured in 10% FBS supplemented with endothelial cell growth supplement (EGM-2 MV; Cambrex Bioscience, Walkersville, MD, USA) [24]. The medium was then replaced with serum free-medium and the confluent HUVECs were treated with human EGF (100 ng/ml in PBS) or human VEGF (AppliChem, St. Louis, MO) (100 ng/ml in PBS) for 6 h. The HUVECs were then harvested and homogenized in TRIzol (Invitrogen, Carlsbad, CA, USA), and the levels of Tie2, Ang1, and Ang2 mRNA were measured by real-time RT-PCR.

### Flow cytometry

Blood was drawn from the tail vein 48 h after reperfusion. The white blood cell fraction, including platelets, was obtained by separation on Ficoll and analyzed by flow cytometry, as previously described [25]. Briefly, cells were labeled with phycoerythrin-labeled anti-VEGFR1 (R&D Systems, MN) and PerCP-Cy5.5-labeled anti-CD11b (LifeSpan Biosciences Inc., WA) antibodies in the presence of an anti-FcR monoclonal antibody (2.4G2; BD Biosciences). After washing, the cells were analyzed in a FACSCalibur flow cytometer (BD Biosciences) and small cells (with low forward scatter [FSC]) were gated for peripheral blood analysis. The percentage of VEGFR1-positive cells was calculated from the flow cytometry results.

### Statistical analysis

All results are expressed as the mean ± standard error of the mean (SEM). All statistical analyses were performed using GraphPad Prism version 5.01 (GraphPad Software, La Jolla, CA). Student's t-test was used for comparisons between two groups. One-way analysis of variance followed by Bonferroni's post-hoc test was used for comparisons between multiple groups. A P-value<0.05 was considered statistically significant.

## Results

### VEGFR1 signaling promotes liver repair after hepatic I/R

**Figure 1:**
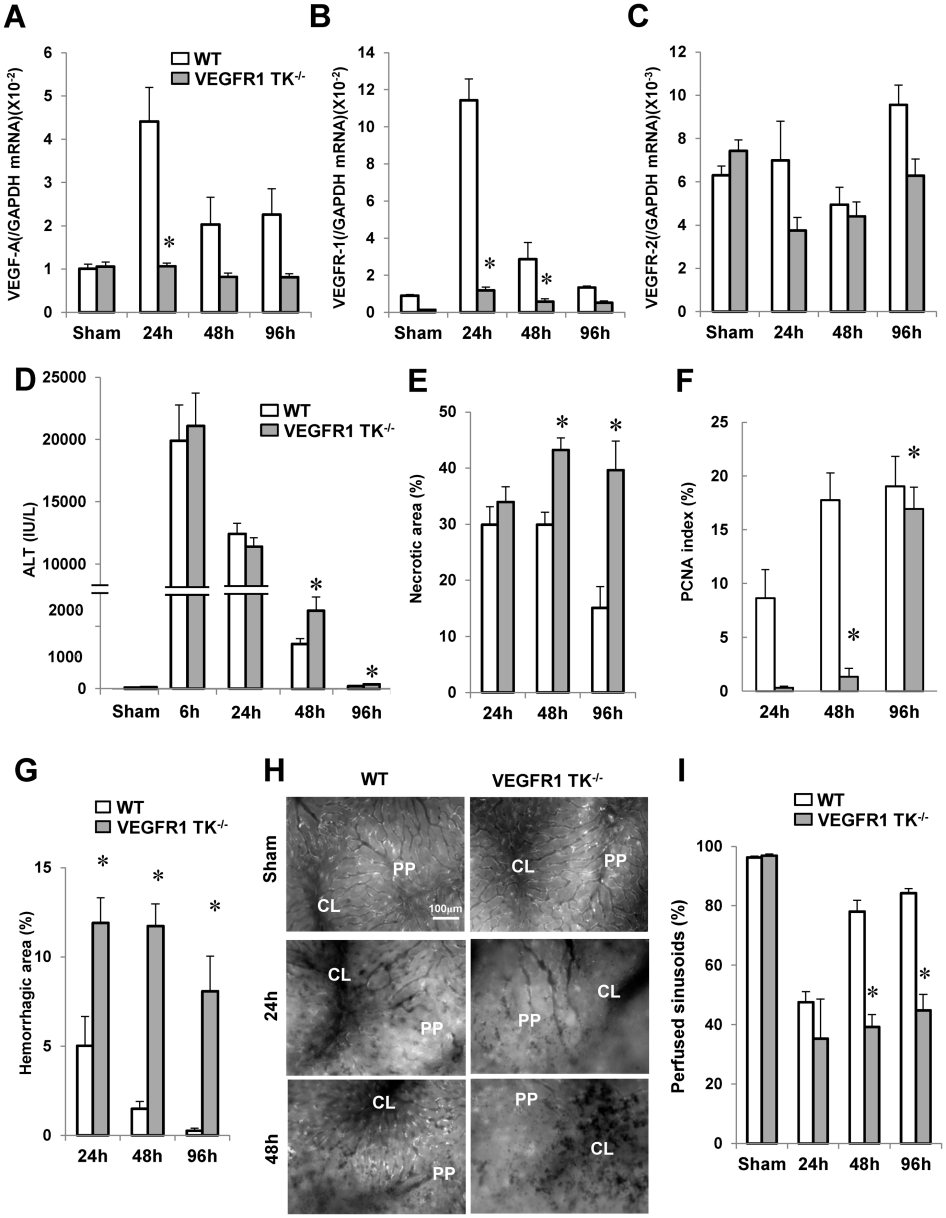
Delayed liver repair and sinusoidal reconstruction after hepatic I/R in VEGFR1 TK^-/-^ mice. Changes in VEGF-A (A), VEGFR1 (B), and VEGFR2 (C) mRNA levels in livers from WT mice and VEGFR1 TK^-/-^ mice after hepatic I/R, and changes in ALT levels (D), the area of hepatic necrosis (E), the PCNA index (F), and the hemorrhagic area (G). Representative *in vivo* microscopic images showing the uptake of acetylated LDL (white dots) at 24 h and 48 h (H). PP, periportal region; CL, centrilobular region. Sinusoidal perfusion after hepatic I/R (I). Data are expressed as the mean ± SEM from five to six mice per group. *p<0.05 *vs*. WT mice.

To investigate the involvement of VEGFR1 in liver repair after hepatic I/R, we determined the expression of VEGF-A/VEGFR1. The levels of VEGF mRNA expression in the livers of WT mice were significantly increased at 24 h after reperfusion by 4.4-fold when compared with sham-controls (p<0.05, n = 5–6 per group) (Fig. 1A). In VEGFR1 TK^-/-^ livers, there were no significant differences in VEGF mRNA levels during the time period of hepatic I/R (p<0.05, n = 5–6 per group). At 24 h after reperfusion, the level of VEGF-A mRNA expression in WT livers was 4.1-fold greater than that in VEGFR1 TK^-/-^ livers (Fig. 1A). VEGFR1 levels in WT livers increased by 9.6-fold, peaking at 24 h before returning to sham control levels by 96 h (n = 5–6 per group) (Fig. 1B). There was no difference in VEGFR2 expression between the two genotypes (n = 5–6 per group) (Fig. 1C). Immunostaining revealed increased expression of VEGFR1 in non-parenchymal cells of WT livers; however, the increase in expression in VEGFR1 TK^-/-^ livers was barely noticeable (Fig. S1).

To examine the importance of VEGFR1 in hepatic I/R injury, we next measured ALT levels (n = 5–6 per group) (Fig. 1D). Both WT mice and VEGFR1 TK^-/-^ mice experienced maximal injury at 6 h. There were no significant differences in ALT levels between the genotypes at 6 h and 24 h; however, ALT levels in VEGFR1 TK^-/-^ mice at 48 h and 96 h were 1.7-fold and 1.6-fold higher, respectively, than those in WT mice. Hepatic necrosis was evident in WT mice at 24 h and 48 h, but was less evident at 96 h (n = 5–6 per group) (Fig. 1E). In VEGFR1 TK^-/-^ mice, extensive necrosis was more evident at 48 h and 96 h (a 1.4- and 2.6-fold increase, respectively, over that in WT mice; Fig. 1E). These results suggest that a lack of VEGFR1 signaling impairs liver repair after hepatic I/R without altering the degree of initial liver injury.

To characterize the regenerative response, liver sections were stained for PCNA, a marker for S-phase of the cell cycle (Fig. S2A). PCNA expression in WT livers increased from 24 h to 96 h (n = 5–6 per group) (Fig. 1F). These results are consistent with previous reports showing that the liver enters a proliferative phase by 48 h post-reperfusion [17], [26]. However, there was no increase in PCNA staining of VEGFR1 TK^-/-^ livers until 96 h (Fig. 1F). We also investigated the expression of pH 3, a marker for M-phase. The expression of pH 3 in WT livers was enhanced at 48 h when compared with that in VEGFR1^-/-^ livers (n = 4 per group) (Fig. S2B).

### Impaired sinusoidal restoration in VEGFR1 TK^-/-^ mice in response to hepatic I/R injury

Next, we asked whether VEGFR1 signaling is involved in sinusoidal injury. The size of the hemorrhagic area within WT livers decreased with time after reperfusion, while that in VEGFR1 TK^-/-^ livers remained high (n = 5–6 per group) (Fig. 1G). A major physiological role of LSEC is scavenging small macromolecules [27]; therefore, we used *in vivo* microscopy techniques to assess the function of LSEC in terms of their ability to take up acetylated low density lipoprotein (LDL) via scavenger receptors (Fig. 1H). In WT mice, uptake was impaired at 24 h, but was restored at 48 h. By contrast, uptake in VEGFR1 TK^-/-^ mice was reduced at 24 h and 48 h. Sinusoidal perfusion in WT mice was reduced by 47% at 24 h (Fig. 1I); however, the rate recovered to 80% of that in controls by 48 h (n = 5–6 per group). In VEGFR1 TK^-/-^ mice, liver microcirculation was disrupted at 24 h and remained impaired at 48 h. At 96 h, sinusoidal perfusion in WT mice was restored to 85% of controls. The rate did not fully reach to that in controls, but there was no significant difference in sinusoidal perfusion rate between 96 h and controls. By contrast, liver microcirculation in VEGFR1 TK^-/-^ mice still remained disturbed at 96 h. These findings suggest that VEGFR1 signaling plays a central role in sinusoidal restoration after hepatic I/R injury.

### VEGFR1 increases the expression of angiogenic factors after hepatic I/R

**Figure 2:**
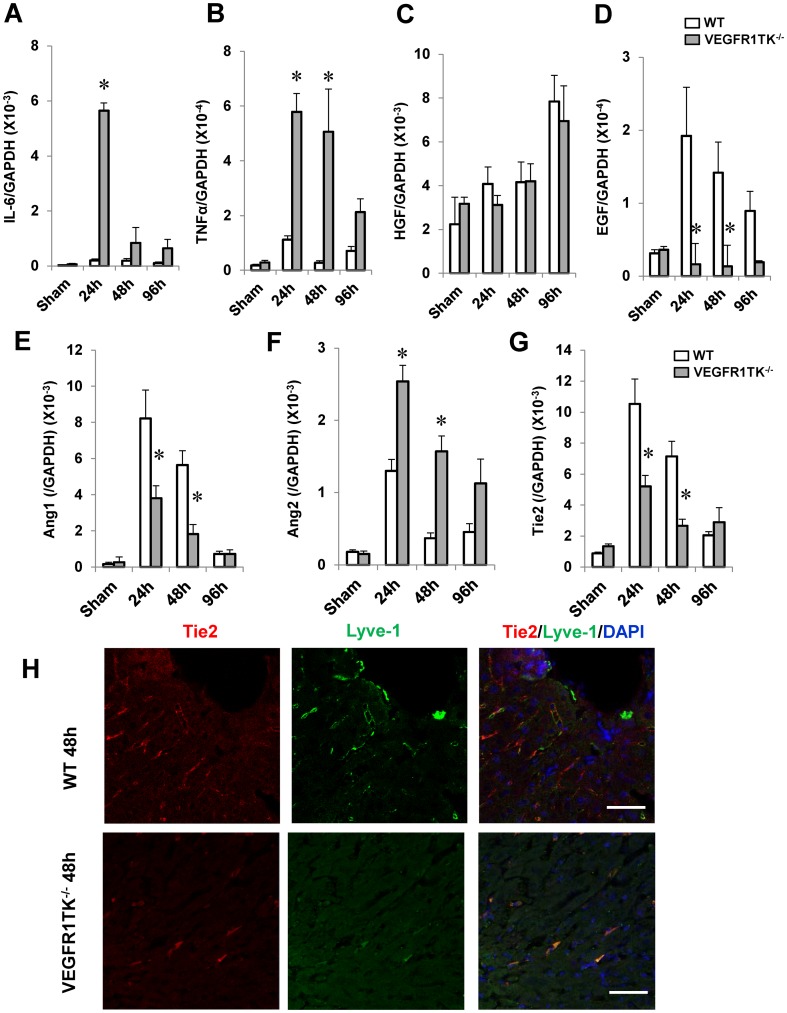
Hepatic expression of growth factors and angiogenic factors in WT and VEGFR1 TK^-/-^ mice after hepatic I/R. (A–D) IL-6 (A), TNFα (B), HGF (C), and EGF (D) mRNA levels in livers from WT mice and VEGFR1 TK^-/-^ mice measured by real-time PCR. Data are expressed as the mean ± SEM from six mice per group. *p<0.05 *vs*. WT mice. (E–G) Levels of Ang1 (E), Ang2 (F), and Tie2 (G) mRNA in livers from WT mice and VEGFR1 TK^-/-^ mice. Data are expressed as the mean ± SEM from six mice per group. *p<0.05 *vs*. WT mice. (H) Double staining of Tie2 and Lyve-1 at 48 h. Tie2 (red) and Lyve-1 (green) colocalize in the sinusoids. Cell nuclei are stained by DAPI (blue). Scale bar, 50 µm.

We measured growth factor expression after hepatic I/R (n = 6 per group). The levels of IL-6 and TNFα mRNA in the liver of both genotypes increased after I/R (Fig. 2A,B). However, the levels in VEGFR1 TK^-/-^ livers were consistently higher than those in WT livers. No significant differences in HGF levels were observed between the genotypes (Fig. 2C). The levels of EGF mRNA in WT livers at 24 h and 48 h increased by 6.1-fold and 4.5-fold, respectively, compared with sham-controls. In VEGFR1 TK^-/-^ livers, there were no significant differences in EGF mRNA levels after reperfusion. Increased EGF mRNA levels in WT livers at 24 h and 48 h were attenuated by approximately 90% in VEGFR1 TK^-/-^ livers (Fig. 2D). These results suggest that VEGFR1 contributes to liver repair by up-regulating EGF.

We next analyzed the expression of pro-angiogenic (Ang1 and its receptor, Tie2) and anti-angiogenic (Ang2) genes, which are necessary for vascular development and angiogenesis [28]. The mRNA levels of Ang1 and Tie2 in WT livers (24 h and 48 h) and in VEGFR1 TK^-/-^ livers (24 h) were increased as compared with respective sham-controls (Fig. 2E,G) (p<0.05, n = 6 per group). The levels of Ang2 mRNA in WT livers (24 h) and in VEGFR1 TK^-/-^ livers (24 h, 48 h, and 96 h) were increased as compared with respective sham-controls (Fig. 2F) (p<0.05, n = 6 per group). The mRNA levels of Ang1 and Tie2 in VEGFR1 TK^-/-^ livers at 24 h and 48 h were lower than those in WT livers. By contrast, those of Ang2 in VEGFR1 TK^-/-^ livers at 24 h and 48 h were further higher than those in WT livers.

Staining for Tie2 was high in the sinusoids during the repair phase (48 h) (Fig. 2H). Lyve-1, a marker for endothelial cells in the liver [16], was diffusely expressed along the sinusoids of sham-controls (Fig. S3A). At 48 h, Lyve-1 was expressed in the injured WT livers, but the expression was scattered as compared with sham-controls (Fig. 2H). Interestingly, Lyve-1 was broadly expressed along the sinusoids of the minimal injured regions of both WT livers and VEGFR1-/- livers (Fig. S3B). These suggested that scattered expression of Lyve-1 indicates the damaged conditions of LSECs during acute liver injury [16]. Double immunofluorescence staining revealed that Tie2 co-localized with Lyve-1 (Fig. 2H), indicating that Tie2 is expressed in LSEC. The expression of Tie2/Lyve-1 in the sinusoids of VEGFR1- TK^-/-^ livers was attenuated compared with WT livers. These results suggest that VEGFR1 plays a critical role in sinusoidal restoration after hepatic I/R injury through increased expression of angiogenic factors.

### VEGFR1 mediates the recruitment of macrophages during hepatic I/R

**Figure 3:**
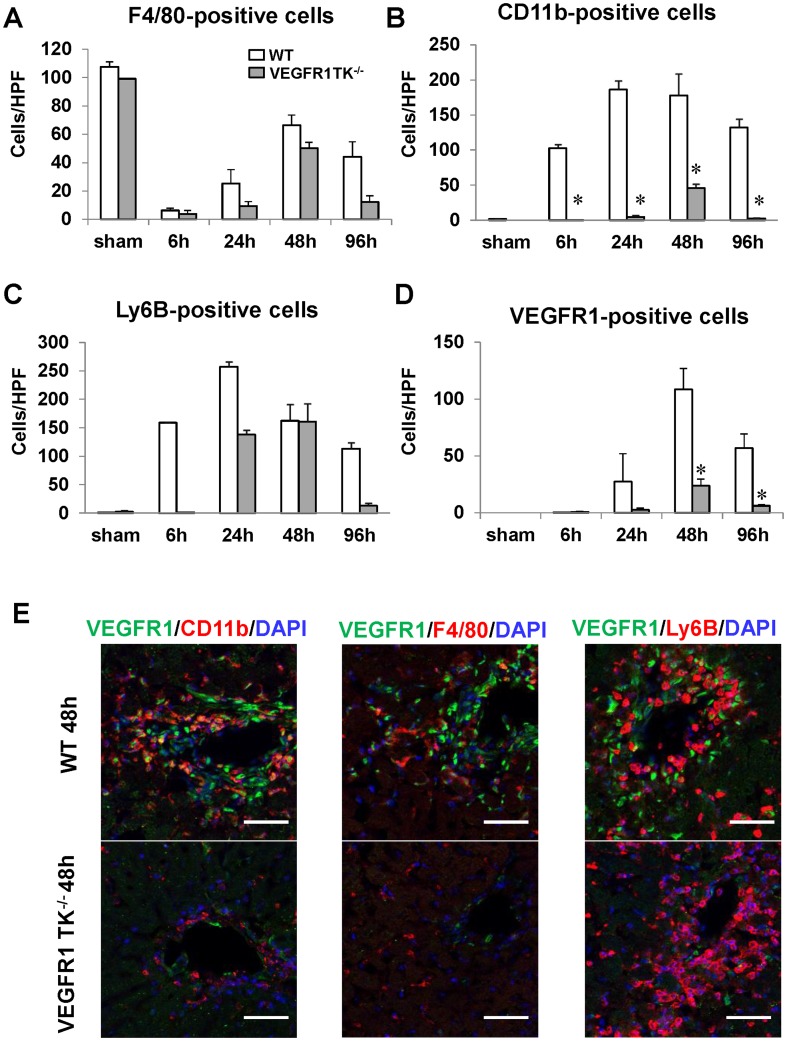
Infiltration of the liver by macrophages from WT and VEGFR1 TK^-/-^ mice after hepatic I/R. (A–D) Changes in the number of F4/80- (A), CD11b- (B), Ly6B- (C), and VEGFR1-positive cells (D) in WT livers and VEGFR1 TK^-/-^ livers after hepatic I/R. Data are expressed as the mean ± SEM from five to six mice per group. *p<0.05 *vs*. WT mice. (E) Double immunostaining of livers from WT mice and VEGFR1 TK^-/-^ mice with antibodies against VEGFR1 (green) and CD11b (red), F4/80 (red) or Ly6B (red) at 48 h post-reperfusion. Hepatocyte nuclei are stained with DAPI (blue). Yellow staining indicates co-localization in double-labeled cells. All images are representative of three independent samples. Scale bars, 50 µm.

Recruited hepatic macrophages play an important role in liver repair after liver injury [29]. Immunostaining revealed that the number of F4/80-positive cells in WT livers and VEGFR1 TK^-/-^ livers reduced compared with sham-controls, reaching a nadir at 6 h and then increasing gradually thereafter (n = 5–6 per group) (Fig. 3A, Fig. S4). Although the number of F4/80-positive cells in WT livers and VEGFR1 TK^-/-^ livers was lower than that in sham-controls at 48 h and 96 h, the difference was not significant (Fig. 3A). By contrast, few CD11b-positive cells were found in sham-controls, whereas marked recruitment of CD11b-positive cells to WT livers was observed from 6 h to 96 h (n = 5–6 per group) (Fig. 3B). CD11b-positive cells in VEGFR1 TK^-/-^ livers were reduced compared to WT livers. Ly6B-positive cells (neutrophils) were recruited to WT livers, whereas these cells accumulated more slowly in the livers of VEGFR TK-/- mice; however, the difference was not significant (n = 5–6 per group) (Fig. 3C). Massive accumulation of VEGFR1-positive cells was noted in WT livers at 48 h, whereas the accumulation was less marked in VEGFR1 TK^-/-^ livers (n = 5–6 per group) (reduced by 80%; Fig. 3D). We performed immunofluorescence double staining for VEGFR1 and CD11b to examine liver cell-specific expression of VEGFR1. The results showed that most of the VEGFR1-positive cells in WT livers were CD11b-positive (Fig. 3E). There was minimal co-localization of VEGFR1 with F4/80 or Ly6B (Fig. 3E). The number of VEGFR1 and CD11b double-positive cells was lower in VEGFR1 TK^-/-^ livers than in WT livers. This indicates that VEGFR1 is likely expressed on recruited macrophages, which is consistent with results previously reported by others [12] and ourselves [16], [17]. Taken together, the results suggest that VEGFR1 signaling mediates the recruitment of VEGFR1-expressing macrophages to the damaged liver.

### EGF secreted by VEGFR1-positive cells facilitates liver repair after hepatic I/R

**Figure 4:**
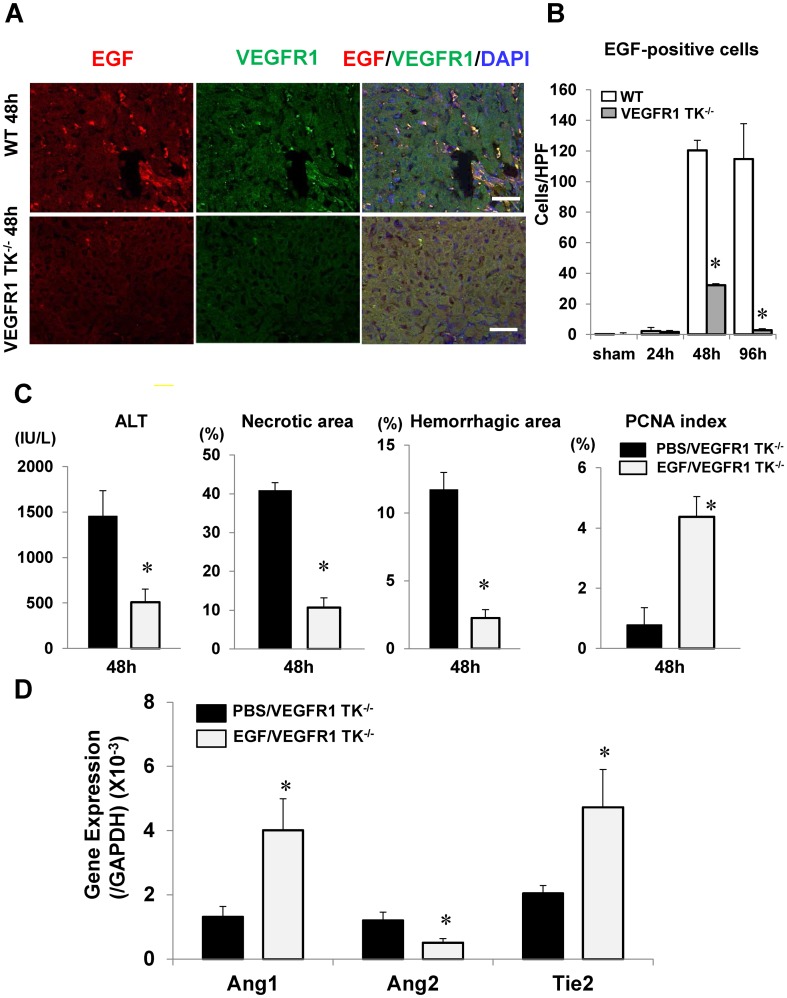
Effect of EGF on liver injury after hepatic I/R. (A) Double immunostaining of mouse livers with antibodies against EGF (red) and VEGFR1 (green) at 48 h post-reperfusion. Scale bar, 50 µm. (B) EGF-positive cells in WT and VEGFR1 TK^-/-^ livers. Data are expressed as the mean ± SEM from five to six mice per group. *p<0.05 *vs*. WT mice. (C,D) Effects of EGF or PBS on liver injury in VEGFR1 TK^-/-^ mice at 48 h post-reperfusion. (C) ALT levels, necrotic area, hemorrhagic area, and PCNA index. (D) Levels of Ang1, Ang2, and Tie2 mRNA in the liver at 48 h post-reperfusion. Data are expressed as the mean ± SEM from five to six mice per group. p<0.05 *vs*. PBS-treated WT mice.

Because increased EGF expression was associated with liver repair (Fig. 2D), we next attempted to identify the cellular source of EGF. Immunofluorescence analysis showed that EGF was expressed in the sinusoids (Fig. 4A). Double immunostaining showed that EGF-positive cells in WT livers were also positive for VEGFR1 (Fig. 4A). Quantitative analysis revealed that the number of EGF-positive cells in WT livers increased at 48 h and 96 h as compared with sham-controls (n = 5–6 per group). In VEGFR1 TK^-/-^ livers, EGF-positive cells were transiently increased at 48 h as compared to controls, returning to the levels of controls by 96 h. The numbers of EGF cells in VEGFR1 TK^-/-^ livers at 48 h and 96 h were lower by 73.3% and 97.5%, respectively, than those in WT livers (Fig. 4B). These results suggest that VEGFR1 facilitates liver repair by inducing EGF production by VEGFR1-expressing macrophages.

To examine the involvement of EGF in liver repair, VEGFR1 TK^-/-^ mice were treated with EGF or PBS (n = 5–6 per group). EGF attenuated liver injury, as indicated by lower levels of ALT, reduced areas of necrosis and hemorrhage, and by higher PCNA expression at 48 h (Fig. 4C). Administration of EGF increased Ang1 and Tie2 mRNA levels, and decreased Ang2 levels (Fig. 4D). The application of EGF to HUVECs *in vitro* increased Tie2 mRNA expression, but not mRNA levels of Ang1 or Ang2 (n = 3 per independent cell isolations) (Fig. S5A). These results indicate that EGF from VEGFR1-expressing macrophages facilitates liver repair and sinusoidal restoration. We also investigated whether VEGF affects the expression of these angiogenic factors. The stimulation of HUVECs with VEGF enhanced the expression of Ang2, but not Ang1 or Tie2 (Fig. S5B).

To evaluate whether EGF expression by VEGFR1-expressing macrophages recruited in the liver is dependent on the VEGF/VEGFR1 pathway, we stimulated isolated peritoneal macrophages from WT and VEGFR1 TK^-/-^ mice with VEGF (n = 3 per independent cell isolations). VEGF increased the levels of EGF, VEGF, and VEGFR1 mRNA in WT macrophages (Fig. S6A–C). By contrast, VEGF had no effect on mRNA levels in VEGFR1 TK^-/-^ macrophages. These results suggest that VEGFR1 signaling in macrophages induces the expression of EGF, VEGF, and VEGFR1.

### VEGFR1-positive macrophages that repair the ischemic liver after I/R are recruited from the BM

**Figure 5:**
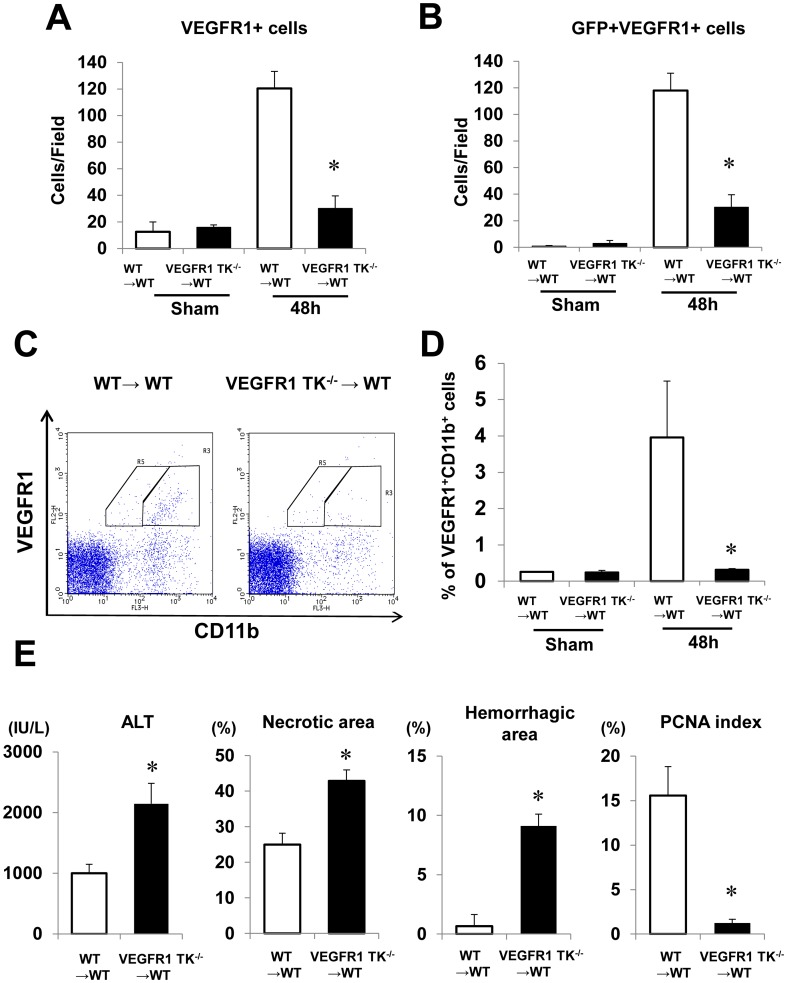
Recruitment of VEGFR1-positive cells from the BM, and hepatic I/R injury in BM chimeric mice. BM cells from GFP+WT mice (WT→WT) or GFP+VEGFR1 TK^-/-^ mice (VEGFR1 TK^-/-^ →WT) were injected into the tail vein of WT mice. (A,B) VEGFR1-positive cells (A) and double-positive cells (GFP- and VEGFR1-positive) (B) in the liver at 48 h post-reperfusion. (C) Expression of VEGFR1 and CD11b in the peripheral blood. Representative flow cytometry plots for GFP+WT BM (left panel) and GFP+VEGFR1 TK^-/-^ BM chimeric mice (right panel) are shown. (D) The percentage of BM-derived VEGFR1/CD11b-positive cells at 48 h at 48 h post-reperfusion.as assessed by FACS analysis. (E) Effect of transplanting BM cells from WT mice and VEGFR1 TK-/- mice on liver repair and hepatocyte proliferation at 48 h post-reperfusion. ALT levels, the area of hepatic necrosis, the hemorrhagic area, and the PCNA index were determined. Data are expressed as the mean ± SEM from six mice per group. *p<0.05 *vs*. GFP+WT BM chimeric mice.

Because BM-derived macrophages contribute to liver repair after acute liver injury [30], [31], we next examined whether recruited VEGFR1-positive cells were derived from the BM. To this end, we generated BM chimeras in which WT mice were transplanted with BM cells from GFP+WT mice (GFP+WT BM chimeric mice) or GFP+VEGFR1 TK^-/-^ mice (GFP+VEGFR1 TK^-/-^ BM chimeric mice) (n = 6 per group). Double immunofluorescence staining revealed that VEGFR1-positive cells were also positive for GFP at 48 h (Fig. S7). Most (97%) of the VEGFR1-positive cells in GFP+WT BM chimeric mice were positive for GFP (Fig. 5A,B). The number of VEGFR1/GFP double-positive cells in GFP+VEGFR1 TK^-/-^ BM chimeric mice was 74.9% lower than that in GFP+WT BM chimeric mice (Fig. 5B). In sham-controls, minimal BM-derived VEGFR1-positive cells were shown in both GFP+WT BM chimeric mice and GFP+VEGFR1 TK^-/-^ BM chimeric mice (Fig. 5B). We also found that the BM-derived VEGFR1 cells were CD11b-positive, and that the number of these cells was lower in GFP+VEGFR1 TK^-/-^ BM chimeric mice than in GFP+WT mice (Fig. S8A). FACS analysis showed that the percentage of circulating VEGFR1/CD11b-positive cells in GFP+VEGFR1 TK^-/-^ BM chimeric mice was reduced compared with that in GFP+WT BM chimeric mice (Fig. 5C,D). These results suggested that VEGFR1 signaling plays a critical role in the mobilization and recruitment of BM-derived VEGFR1-positive macrophages. Furthermore, in sham-controls, there was no significant difference in the percentage of circulating VEGFR1/CD11b-positive cells in both GFP+WT BM chimeric mice and GFP+VEGFR1 TK^-/-^ BM chimeric mice (Fig. 5D). These suggested that the mobilization of BM cells is not impaired in both BM chimeric mice and BM cells pools are not reduced in both mice.

We next examined whether VEGFR1 signaling in BM-derived cells affected liver injury. ALT levels and the areas of necrosis and hemorrhage in the livers of GFP+VEGFR1 TK^-/-^ BM chimeric mice increased at 48 h after hepatic I/R, whereas the PCNA index decreased, compared with those in GFP+/WT BM chimeric mice (Fig. 5E). The levels of EGF, Ang1, and Tie2 mRNA were lower in GFP+VEGFR1 TK^-/-^ BM chimeric mice than those in GFP+/WT BM chimeric mice, and Ang2 levels in GFP+VEGFR1 TK^-/-^ BM chimeric mice were higher than those in GFP+/WT BM chimeric mice (Fig. S8B). VEGFR1 levels but not VEGF and VEGFR2, were also lower in GFP+VEGFR1 TK^-/-^ BM chimeric mice than those in GFP+/WT BM chimeric mice (Fig. S8C). These results suggest VEGFR1-expressing BM cells contribute to liver repair and sinusoidal reconstruction.

Finally, we investigated whether BM-derived VEGFR1-positive cells express EGF at 48 h after reperfusion (Fig. S8D). The results showed that GFP/VEGFR1-positive cells did express EGF. The number of BM-derived VEGFR1/EGF cells was lower in GFP+/+VEGFR1 TK-/- BM chimeric mice than that in GFP+WT BM chimeric mice. These results suggest that VEGFR1 signaling in BM cells promotes liver repair through increased EGF expression by VEGFR1-expressing cells recruited from the BM.

## Discussion

The liver possesses a remarkable ability to regenerate after acute injury; however, the molecular mechanisms underlying liver recovery from hepatic I/R injury remain unclear. Recent studies identified novel participants in liver repair. Signaling through CXC chemokines and their receptors, CXCR1 and CXCR2, in hepatocytes [5], and serotonin released from platelets [4] are important mediators that regulate liver repair. A murine model of partial hepatectomy, in which remnant hepatocytes are intact, suggested a role for VEGFR2 in liver regeneration [14]. Also, VEGFR1 is critical for liver repair in chemically-induced models of liver injury, in which hepatocytes and LSEC are severely injured [15], [16]. Here, we found that VEGFR1 is essential for liver repair after hepatic I/R injury. VEGFR1 signaling recruits VEGFR1-expressing EGF-producing macrophages, which are involved in repairing the sinusoids by inducing pro-angiogenic gene expression.

Macrophage recruitment is essential for liver repair after toxin-induced acute injury [29]–[31]. We previously showed that macrophage accumulation during hepatotoxicity is necessary for the repair of the liver and associated microvasculature [16], [32]. Recent evidence suggests that VEGFR1 mediates monocyte/macrophage infiltration to local inflammatory sites [8], [12], and that VEGFR1 promotes the recruitment of VEGFR1-expressing macrophages to repair acetaminophen-induced liver injury [16]. The recruitment of peritoneal macrophages is dependent on VEGFR1 signaling [8], and VEGF induces chemotaxis in peritoneal macrophages through VEGFR1-mediated mechanisms [33]. Together with the results reported herein, these findings indicate that signaling through VEGFR1 is important for the recruitment of VEGFR1-expressing macrophages to repair I/R-induced liver injury.

The macrophages recruited to the liver in response to hepatotoxicity are derived from the BM [30], [31]. The present study also suggests that VEGFR1-expressing macrophages are derived from the BM, and that the recruitment of BM-derived VEGFR1- expressing macrophages to the injured livers after hepatic I/R is dependent of VEGFR1 signaling, which is consistent with our recent results in a model of murine gastric ulcer healing [18] and wound healing [13]. VEGFR1 signaling in BM cells are crucial for not only the recruitment of VEGFR1-macrophages, but also the mobilization of VEGFR1-macrophages into circulation (Figure 5D). Therefore, suppressed mobilization of VEGFR1-positve cells results in attenuated recruitment of VEGFR1-positive cells into the liver. We also have shown that VEGFR1 signaling is important for the mobilization of VEGFR1-positive cells into circulation and subsequent recruitment of these cells into gastric ulcer granulation tissue to promote the ulcer healing process [18]. Furthermore, enhanced hepatic levels of VEGF, a ligand of VEGFR1, could be responsible for recruitment of VEGFR1 cells into the livers. VEGF is known to recruit VEGFR1-expressing macrophages [34] and bone marrow-derived macrophages [13]. Collectively, the recruitment of BM-derived VEGFR1-expressing macrophages into the injured livers is at least partly mediated by VEGF/VEGFR1 signaling pathway, and VEGFR1 signaling in BM cells appears to contribute to liver repair and sinusoidal reconstitution after hepatic I/R; thus VEGFR1 signaling promotes the recruitment of VEGFR1-positive macrophages during the repair phase of hepatic I/R injury.

In the present study, F4/80 and CD11b were used to identify tissue-resident macrophages (Kupffer cells) and tissue-infiltration macrophages, respectively, by immunofluorescence [17]. However, a single macrophage marker is not enough to distinguish resident Kupffer cells and recruited hepatic or peritoneal macrophages [30], [31], [35]. Flow cytometric analysis by Kinoshita et al. [34] reveals that murine F4/80-positive resident Kupffer cells could be classified into two subsets, cytokine-producing CD11b-positive cells and phagocytic and reactive oxygen species (ROS)-producing CD68-positive cells. These findings suggest that F4/80-positive Kupffer cells could be functionally classified into two sub-groups and that F4/80-positive Kupffer cells consist of, at least in part, CD11b-positive cells. Although, the origins of two populations remain uncertain in their study, they speculate that CD11b-positive cells appear to be infiltrated hepatic macrophages and CD68-positive cells to be resident hepatic macrophages [35]. This also suggests that resident Kupffer cells partly overlap the characteristics of infiltrating macrophages. Collectively, the distinction between resident Kupffer cells and recruiting hepatic macrophages is difficult due to the lack of distinctive phenotypical markers, and functional characterization and classification of murine Kupffer cells have yet to be fully elucidated.

Immunofuorescene analysis revealed the reduction in F4/80-positive cells during hepatic I/R injury. Similar findings to our results have been reported in a model of acute liver injury elicited by acetaminophen, demonstrating that F4/80-positive cells are reduced [16], [36]. These findings suggest that Kupffer cells play a minor role in hepatic I/R injury mediated by VEGF/VEGFR1 pathway. However, the results that WT mice indeed exhibited significant hepatic I/R injury do not exclude Kupffer cells as the main mediator of the pathology of hepatic I/R injury [1]. In addition, mice deleted F4/80-positive cells with clodronate liposome are susceptible to hepatic I/R injury [37] as well as hepatotoxicity elicited by acetaminophen [29] and carbon tetrachloride (CCl_4_) [38]. Furthermore, reduction in F4/80-positive Kupffer cells does not indicate that all Kupffer cells are decreased. Despite reduction in F4/80-positive Kupffer cells during CCl_4_ heptotoxicty, increased CD11b-positive Kupffer cells are responsible for induction of acute liver injury [38]. In this regard, Kupffer cells expressing CD11b may be involved in hepatic I/R injury in our model. These findings indicate heterogeneity of Kupffer cells under pathological conditions in which recruited hepatic macrophages are observed.

Following hepatic I/R, VEGF is expressed in the hepatic infiltrating cells [39], which are positive for CD11b, but are negative for myeloperoxidase, suggesting that the source of VEGF during hepatic I/R appears to be CD11b-positive macrophages. Enhanced expression of VEGF/VEGFR1 in the livers would be involved in hepatic I/R injury, though many mediators other than VEGF/VEGFR1 pathway also contribute to the injury. For example, proinflammatory cytokines including IL-6 and TNFα are critical for acute liver injury elicited by hepatic I/R [1]. The results of the present study demonstrated that hepatic I/R up-regulates the expression of IL-6 and TNFα in VEGFR1^-/-^ livers, which is associated with enhanced and sustained hepatic necrosis in VEGFR1^-/-^ livers. In addition, Kupffer cells and newly recruited neutrophils produce reactive oxygen species (ROS) in response to damage signals released from injured hepatocytes, leading to hepatic necrosis during hepatic I/R [1].

Liver regeneration is controlled by several mediators, including cytokines and growth factors [40]. EGF plays a critical role in the proliferative response accompanying liver regeneration after partial hepatectomy [40]. The current data suggest that EGF, secreted by VEGFR1-expressing macrophages, promotes liver repair after hepatic I/R injury. Indeed, treating WT mice with an anti-EGF antibody delays hepatocyte proliferation and liver repair after hepatic I/R [17]. VEGFR1 signaling increases EGF and VEGF expression in VEGF-stimulated macrophages [12]. EGF expression is increased in VEGFR1/CD11b-positive cells within gastric ulcer granulation tissues during gastric ulcer healing [18]. This suggests that stimulating VEGFR1 on macrophages might serve to amplify EGF expression to repair I/R-induced liver injury. Furthermore, VEGFR1 signaling in BM cells promotes liver repair through enhancement of EGF in BM-derived VEGFR1-expressing macrophages recruited to injured livers during hepatic I/R. To confirm this, further experimental studies of VEGFR1^-/-^ mice with BM cells transplanted from WT mice will be necessary.

VEGFR1 signaling also protects LSEC from injury and promotes functional and structural recovery from I/R-induced damage, as evidenced by the improved endocytic activity of LSEC and reduced areas of hemorrhage. Alternatively, lack of VEGFR1 signaling fails to restore the LSEC structure and function after hepatic I/R injury, which is consistent with our recent results [16]. Additionally, it seems likely that sinusoidal damage and the accumulation of VEGFR1-postive cells closely correlated in hepatic I/R injury. Impaired hepatic microvascular repair reduces the oxygen supply to hepatic tissue, resulting in delayed hepatocyte proliferation. Since LSEC function as scavenging cells that clear circulating waste molecules, including pathogenic acylated or glycosylated proteins [27], suppression of LSEC scavenging during hepatic I/R may inhibit the functional recovery of regenerating hepatocytes.

Loss of VEGFR1 expression in endothelial cells leads to reduced sprout formation and cell migration, which results in reduced vascular branching [41]. VEGFR-1 is thought to positively regulate angiogenesis in other pathological conditions [8]–[10]. In addition, VEGFR1 TK^-/-^ mice show reduced angiogenesis in parallel with decreased recruitment of VEGFR1-expressing macrophages [13]. These findings imply that the VEGFR1 signaling pathway plays an important role in recruiting VEGFR1-expressing macrophages, which promote reconstitution of damaged sinusoids after hepatic I/R injury. By contrast, Ho et al [42] report that knocking out VEGFR1 in postnatal and adult mice increased angiogenesis after cardiac ischemia, which increased the bioavailability of VEGF-A for binding to VEGFR2, markedly increased the expression of VEGFR-2 protein, and promoted signaling downstream of VEGFR2. Thus, the role of VEGFR1 signaling in hepatic tissue remodeling appears to be dependent on the type of organ injury.

Infiltrating macrophages play a critical role not only in sinusoidal recovery from acute liver injury, but also in angiogenesis related to liver fibrosis in chronic liver injury. For instance, CCL2-dependent infiltrating macrophages derived from BM into the injured liver facilitate angiogenesis during the evolution of liver fibrosis through releasing pro-angiogenic factors including VEGF [43]. Additionally, inflammatory hepatic macrophages are involved in angiogenesis with enhancement of VEGF in the progression of nonalcoholic steatohepatitis [44]. Thus, recruited macrophages are likely main mediators of sinusoidal reconstitution and hepatic angiogenesis both in acute and chronic liver injury.

During the repair of hepatic I/R injury, VEGFR1 signaling facilitates sinusoidal restoration via EGF, which is secreted by recruited VEGFR1-expressing macrophages. EGF rescues the hepatic microvasculature from hepatic I/R-induced injury by increasing the expression of pro-angiogenic factors such as Ang1 and Tie2, which are necessary for vascular development and angiogenesis [28]. Concomitantly, preliminary studies showed that an EGF-neutralizing antibody attenuates hepatic expression of Ang1 and Tie2 after hepatic I/R (data not shown). EGF induces angiogenesis and tube formation [45], and enhances Tie2 expression, but neither Ang1 nor Ang2 in HUVECs. Furthermore, involvement of VEGF in Ang2 expression in HUVECs, but neither Ang1 nor Tie2, which is consistent with others [46], suggests that Tie2 expression in ECs is likely regulated by EGF, but not by VEGF. However, caution should be taken in interpretation of the data from HUVECs experiment, because the phenotype and morphology of HUVECs are different from those of LSECs. Ang1 and Tie2 are involved in reconstructing the sinusoids in response to CCl_4_-induced hepatotoxicity [47]. Also, Ang1 induces angiogenesis during wound healing and minimizes renal microvascular injury [48]. With respect to Ang2, the mRNA expression of Ang2 in WT livers is principally enhanced during hepatic I/R. Although Ang2 acts as an antagonist of Ang1, Hu el al [49] have shown that LSEC-derived Ang2 is found to be enhanced during the angiogenic phase of liver regeneration after partial hepatectomy in mice. Their study suggests that Ang2 derived from LSECs as well as Ang1 derived from hepatic stellate cells (HSCs), are required for hepatic angiogenesis during liver regeneration. Therefore, it is plausible that enhanced hepatic expression of Ang2 as well as of Ang1 would contribute to the repair of the hepatic microvasculature after hepatic I/R. In addition, the current study shows that further enhanced expression of Ang2 in VEGFR1^-/-^ livers in comparison with WT livers, suggesting that VEGFR1 signaling down-regulates the expression of Ang2. Intriguingly, higher levels of Ang2 in plasma and in the injured livers are associated with the development of multiple organ dysfunction syndrome and poor outcome in patients with acute liver failure [50]. In this regard, the balance between Ang1 and Ang2 might be important for the sinusoidal reconstitution after hepatic I/R. However, the mechanisms by which VEGFR1 signaling and the Ang-Tie system interact to repair damaged sinusoids after hepatic I/R injury still need to be elucidated.

In conclusion, VEGFR1 signaling is essential for liver repair and sinusoidal reconstruction after hepatic I/R. VEGFR1-dependent recruitment of VEGFR1-expressing macrophages from the BM to the injured liver contributes to sinusoidal reconstruction after hepatic I/R. These macrophages secrete EGF and enhance the expression of pro-angiogenic genes, which in turn promotes liver repair and recovery from hepatic I/R injury. Thus, VEGFR1 activation represents a potential therapeutic strategy to facilitate hepatocellular and sinusoidal repair after acute liver injury.

## Supporting Information

Figure S1 **Representative photographs showing immunofluorescence staining of VEGFR1 in liver sections from WT mice (upper panel) and VEGFR1 TK^-/-^ mice (lower panel) after hepatic I/R. Bar, 50 µm.**
(TIF)

Figure S2 **(A) Representative photographs showing immunohistochemical staining of PCNA in liver sections from WT mice (left panel) and VEGFR1 TK^-/-^ mice (right panel) at 48 h post-reperfusion.** Scale bar, 100 µm. (B) Representative photographs showing immunofluorescence staining of phosphorylated histone H3 (pH 3) in liver sections from WT mice (left panel) and VEGFR1 TK^-/-^ mice (middle panel) at 48 h post-reperfusion. Scale bar, 100 µm. The pH 3 index (right panel). Data are expressed as the mean ± SEM from four mice per group. *p<0.05 *vs*. WT mice.
(TIF)

Figure S3 **(A) Double staining of Tie2 (red) and Lyve-1 (green) in WT livers from sham-controls.** Cell nuclei are stained by DAPI (blue). Scale bar, 50 µm. (B) Immunofluorescent staining of liver sections with Lyve-1 from WT mice and VEGFR^-/-^ mice at 48 h post-reperfusion. Note diffuse expression in the sinusoids of minimal injured regions, and down-regulated expression in injured regions within the WT livers and VEGFR^-/-^ livers subjected to hepatic I/R. I, injured regions. Scale bar, 50 µm.
(TIF)

Figure S4 **Representative photographs showing immunofluorescence staining of F4/80 in liver sections from WT mice after hepatic I/R.** Cell nuclei are stained by DAPI (blue). Bar, 100 µm.
(TIFF)

Figure S5 **Effect of EGF or VEGF on Ang1, Ang2, and Tie2 expression in HUVECs (A,B).** HUVECs were treated with EGF (100 ng/ml) (A) or VEGF (100 ng/ml) (B), and the levels of Ang1, Ang2, and Tie2 mRNA were determined by real-time RT-PCR 4 hours later. Data are expressed as the mean ± SEM of three separate experiments. *p<0.05 *vs*. vehicle (Veh).
(TIF)

Figure S6 **Effect of VEGF on the expression of EGF, VEGF, and VEGFR1 in peritoneal thioglycollate-induced macrophages from WT mice and VEGFR1 TK^-/-^ mice.** (A–C) Isolated macrophages were treated with VEGF, and the levels of EGF (A), VEGF-A (B), and VEGFR1 (C) mRNA were determined by real-time quantitative RT-PCR 6 h later. Data are expressed as the mean ± SEM of three independent experiments. *p<0.05 *vs*. WT mice.
(TIF)

Figure S7 **Typical appearance of GFP+VEGFR1+ cells in the livers of GFP+WT BM (upper panel) and GFP+VEGFR1 TK^-/-^ BM chimeric mice (lower panel) at 48 h post-reperfusion.** Liver tissues from GFP+WT BM and GFP+VEGFR1 TK^-/-^ BM chimeric mice were stained with antibodies against GFP (green) and VEGFR1 (red). Yellow staining indicates co-localization of GFP withVEGFR1. Scale bar, 50 µm.
(TIF)

Figure S8 **Expression of growth factors and angiogenic factors in the livers of WT mice transplanted with BM cells from GFP+WT mice or GFP+VEGFR1 TK^-/-^ mice.** (A) Representative images of liver tissue from GFP+WT BM chimeric mice and GFP+VEGFR1 TK^-/-^ BM chimeric mice at 48 h after hepatic I/R. GFP-positive cells (green) co-expressing VEGFR1 (blue) and CD11b (red) are shown. Arrows indicate triple-positive cells. Scale bar, 25 µm. (B) The levels of EGF, Ang-1, Ang-2, and Tie-2 mRNA in livers from GFP+WT BM and GFP+VEGFR1 TK^-/-^ BM chimeric mice as determined by real-time PCR. Data are expressed as the mean ± SEM from five to six mice per group. *p<0.05 *vs*. GFP+WT BM chimeric mice. (C) The levels of VEGF-A, VEGFR1, and VEGFR2 mRNA were measured by real-time PCR. Data are expressed as the mean ± SEM from four mice per group. *p<0.05 *vs*. GFP+WT BM chimeric mice. (D) Representative photographs of immunofluorescence staining of GFP, VEGFR1, and EGF in mouse livers after hepatic I/R. Liver tissues from GFP+WT BM and GFP+VEGFR1 TK^-/-^ BM chimeric mice were stained with antibodies against GFP (green), VEGFR1 (blue), and CD11b (red) at 48 h post-reperfusion. Merged images are shown. Images are representative of three independent samples. Arrows indicate triple-positive cells. Scale bar, 25 µm.
(TIF)

Table S1 **Primers for real-time RT-PCR.**
(TIF)
