# Supplementary figures and images for: VEGFR1-Positive Macrophages Facilitate Liver Repair and Sinusoidal Reconstruction after Hepatic Ischemia/Reperfusion Injury
Source: PLoS One. 2014 Aug 27;9(8):e105533. doi: 10.1371/journal.pone.0105533 (PMC4146544; doi:10.1371/journal.pone.0105533)

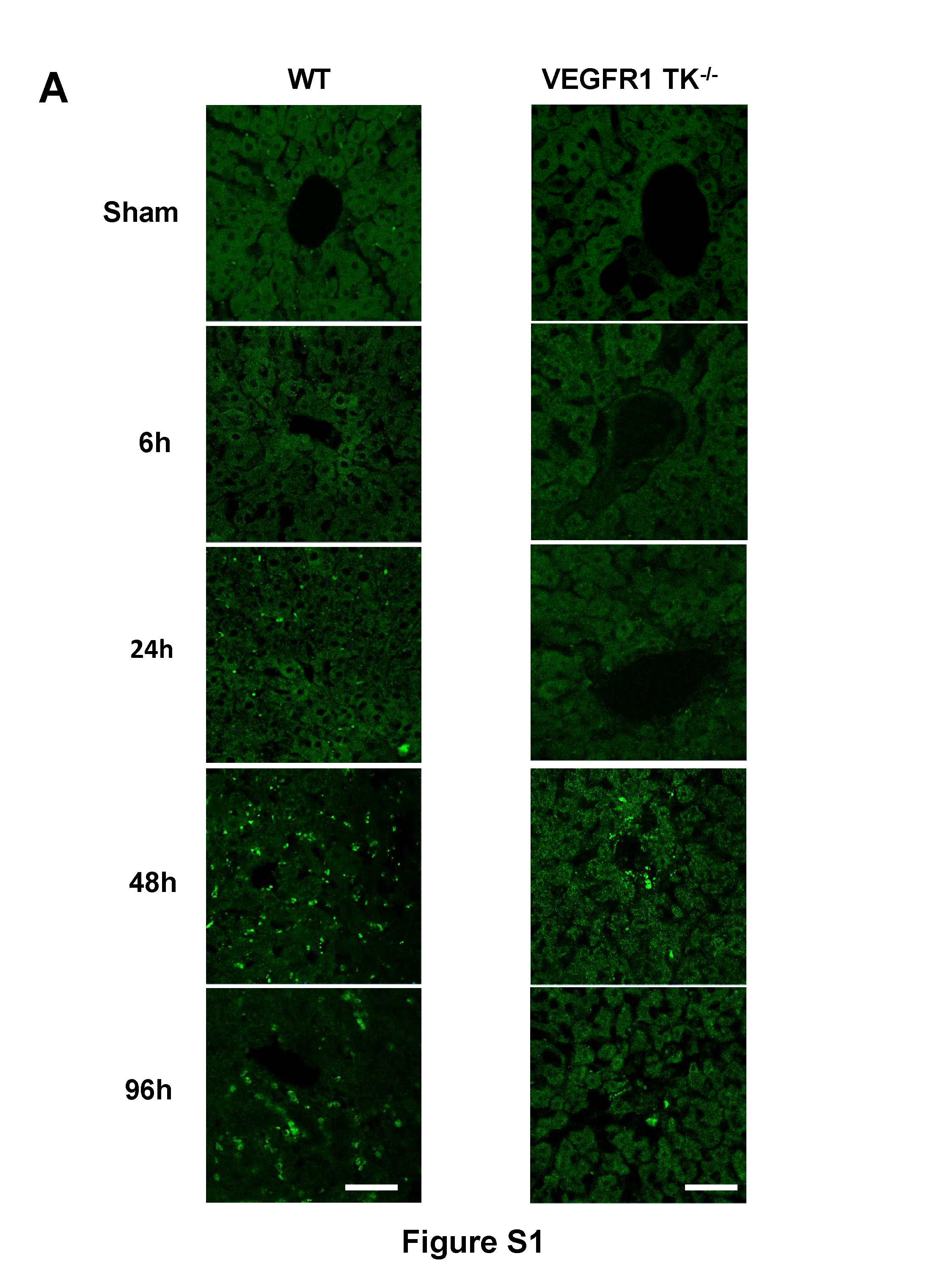

Supplement: Figure S1 — Representative photographs showing immunofluorescence staining of VEGFR1 in liver sections from WT mice (upper panel) and VEGFR1 TK-/- mice (lower panel) after hepatic I/R. Bar, 50 µm. (TIF) [file pone.0105533.s001.tif]

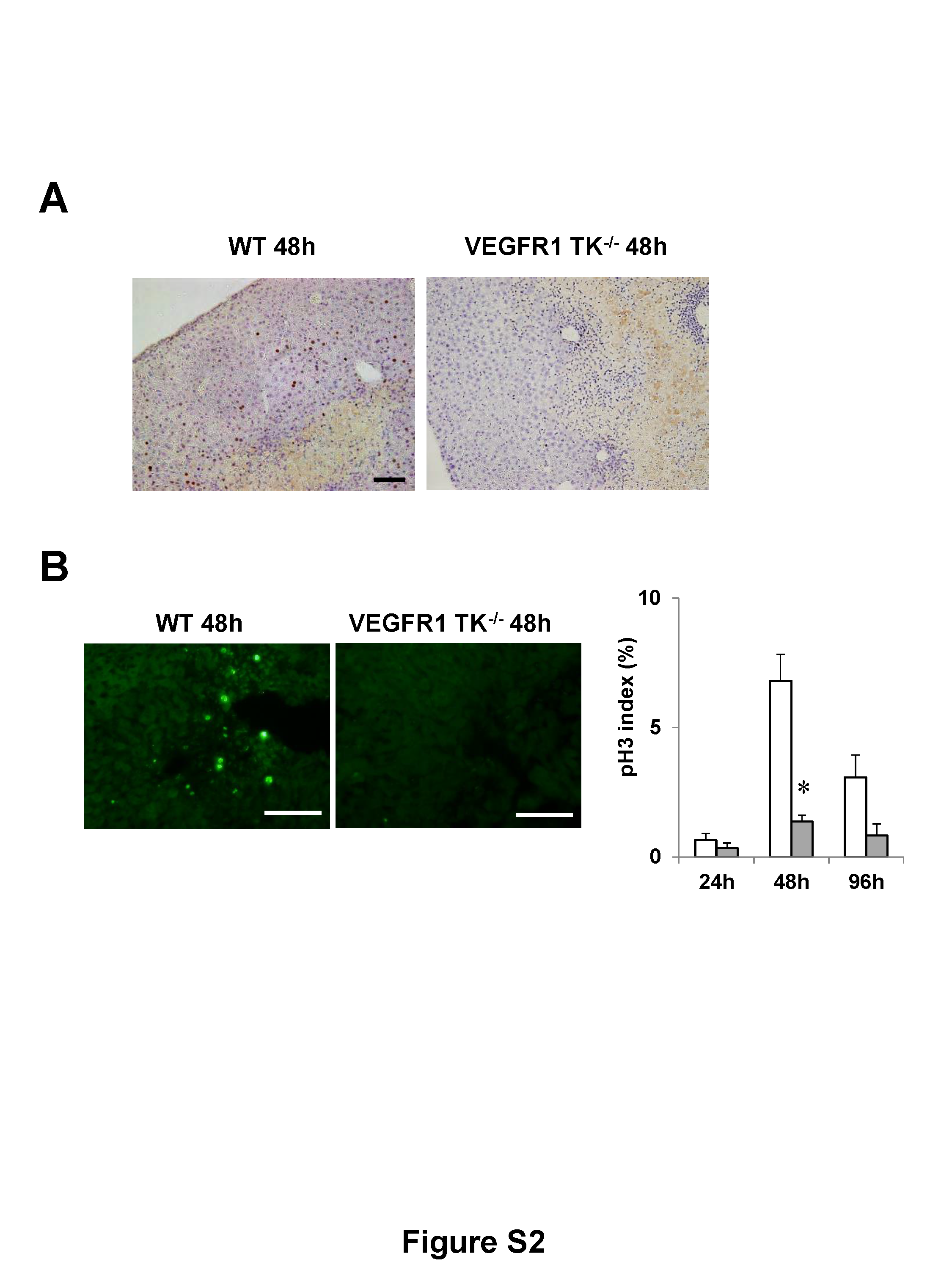

Supplement: Figure S2 — (A) Representative photographs showing immunohistochemical staining of PCNA in liver sections from WT mice (left panel) and VEGFR1 TK-/- mice (right panel) at 48 h post-reperfusion. Scale bar, 100 µm. (B) Representative photographs showing immunofluorescence staining of phosphorylated histone H3 (pH 3) in liver sections from WT mice (left panel) and VEGFR1 TK-/- mice (middle panel) at 48 h post-reperfusion. Scale bar, 100 µm. The pH 3 index (right panel). Data are expressed as the mean ± SEM from four mice per group. *p<0.05 vs. WT mice. (TIF) [file pone.0105533.s002.tif]

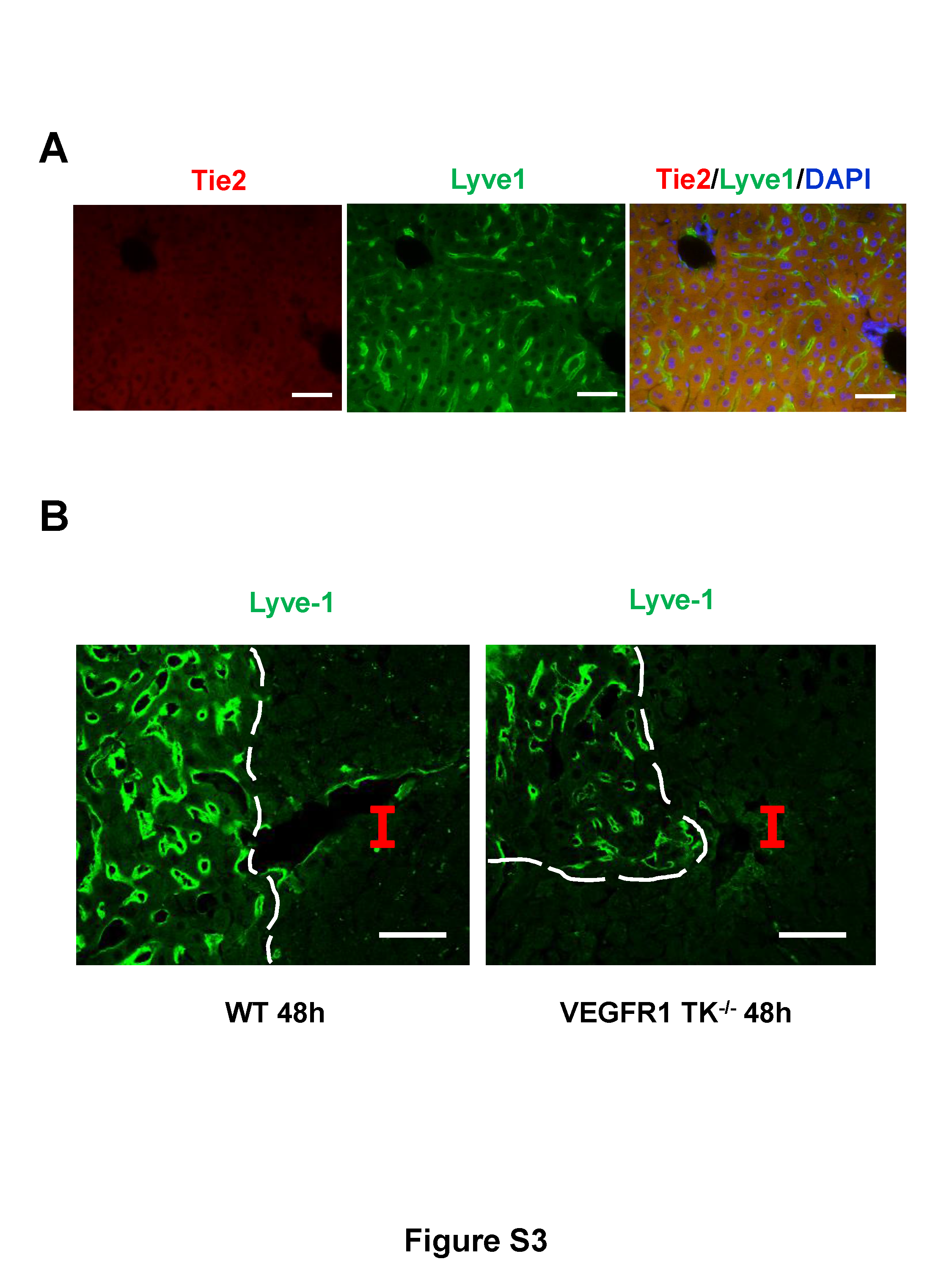

Supplement: Figure S3 — (A) Double staining of Tie2 (red) and Lyve-1 (green) in WT livers from sham-controls. Cell nuclei are stained by DAPI (blue). Scale bar, 50 µm. (B) Immunofluorescent staining of liver sections with Lyve-1 from WT mice and VEGFR-/- mice at 48 h post-reperfusion. Note diffuse expression in the sinusoids of minimal injured regions, and down-regulated expression in injured regions within the WT livers and VEGFR-/- livers subjected to hepatic I/R. I, injured regions. Scale bar, 50 µm. (TIF) [file pone.0105533.s003.tif]

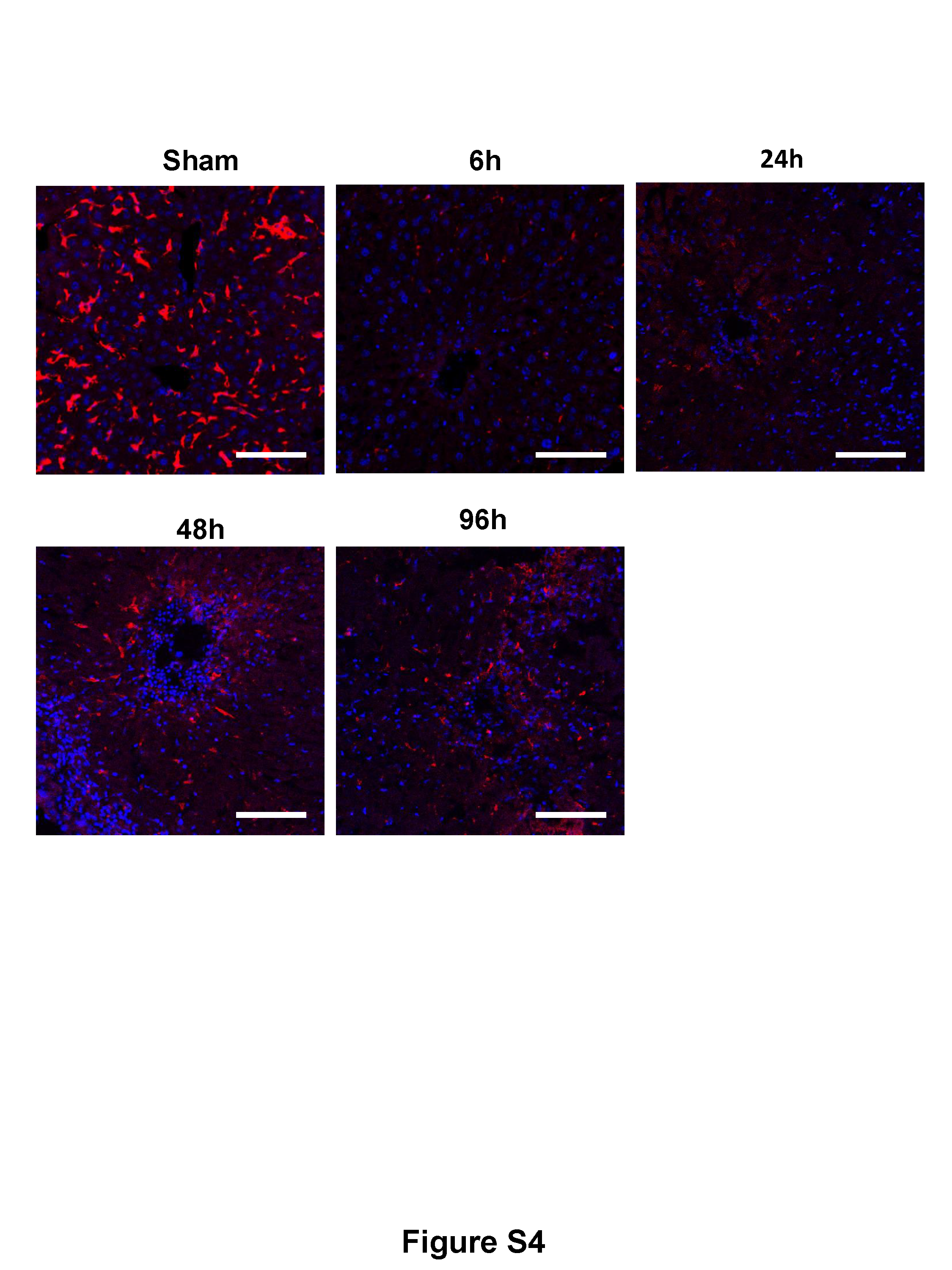

Supplement: Figure S4 — Representative photographs showing immunofluorescence staining of F4/80 in liver sections from WT mice after hepatic I/R. Cell nuclei are stained by DAPI (blue). Bar, 100 µm. (TIFF) [file pone.0105533.s004.tiff]

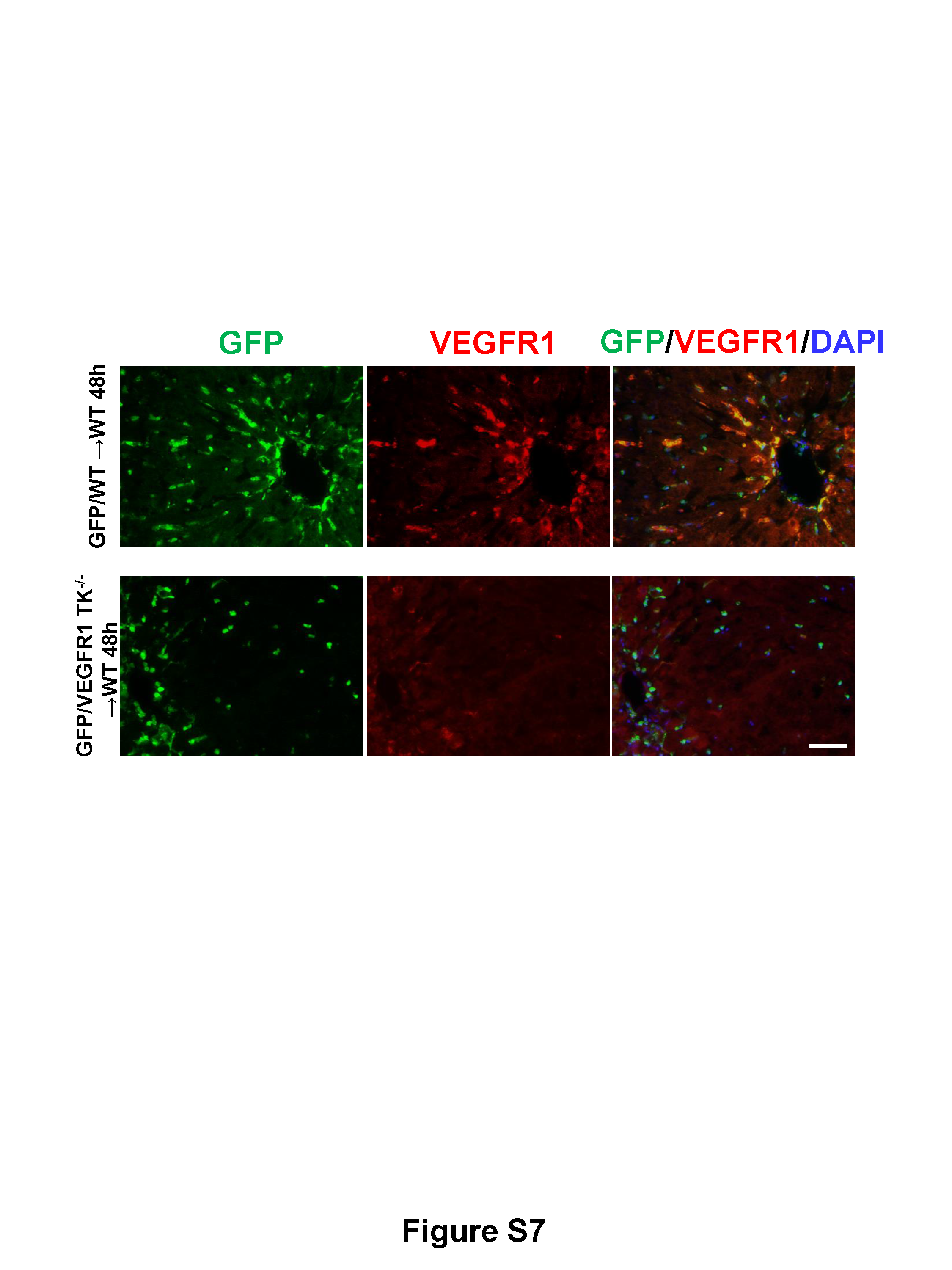

Supplement: Figure S7 — Typical appearance of GFP+VEGFR1+ cells in the livers of GFP+WT BM (upper panel) and GFP+VEGFR1 TK-/- BM chimeric mice (lower panel) at 48 h post-reperfusion. Liver tissues from GFP+WT BM and GFP+VEGFR1 TK-/- BM chimeric mice were stained with antibodies against GFP (green) and VEGFR1 (red). Yellow staining indicates co-localization of GFP withVEGFR1. Scale bar, 50 µm. (TIF) [file pone.0105533.s007.tif]

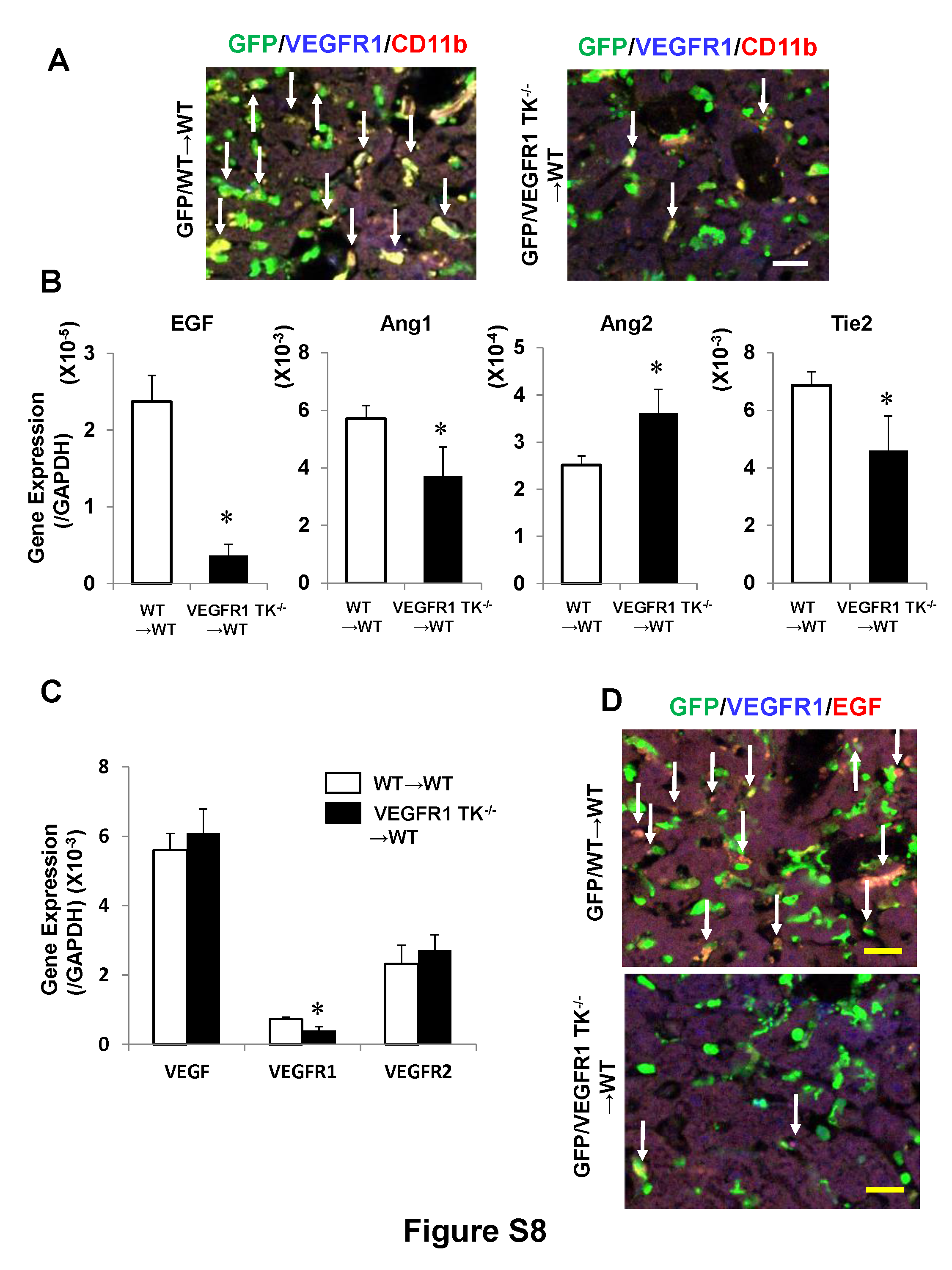

Supplement: Figure S8 — Expression of growth factors and angiogenic factors in the livers of WT mice transplanted with BM cells from GFP+WT mice or GFP+VEGFR1 TK-/- mice. (A) Representative images of liver tissue from GFP+WT BM chimeric mice and GFP+VEGFR1 TK-/- BM chimeric mice at 48 h after hepatic I/R. GFP-positive cells (green) co-expressing VEGFR1 (blue) and CD11b (red) are shown. Arrows indicate triple-positive cells. Scale bar, 25 µm. (B) The levels of EGF, Ang-1, Ang-2, and Tie-2 mRNA in livers from GFP+WT BM and GFP+VEGFR1 TK-/- BM chimeric mice as determined by real-time PCR. Data are expressed as the mean ± SEM from five to six mice per group. *p<0.05 vs. GFP+WT BM chimeric mice. (C) The levels of VEGF-A, VEGFR1, and VEGFR2 mRNA were measured by real-time PCR. Data are expressed as the mean ± SEM from four mice per group. *p<0.05 vs. GFP+WT BM chimeric mice. (D) Representative photographs of immunofluorescence staining of GFP, VEGFR1, and EGF in mouse livers after hepatic I/R. Liver tissues from GFP+WT BM and GFP+VEGFR1 TK-/- BM chimeric mice were stained with antibodies against GFP (green), VEGFR1 (blue), and CD11b (red) at 48 h post-reperfusion. Merged images are shown. Images are representative of three independent samples. Arrows indicate triple-positive cells. Scale bar, 25 µm. (TIF) [file pone.0105533.s008.tif]
